# Carbonization and Preparation of Nitrogen-Doped Porous Carbon Materials from Zn-MOF and Its Applications

**DOI:** 10.3390/ma13020264

**Published:** 2020-01-07

**Authors:** Kulandaivel Sivasankar, Souvik Pal, Murugan Thiruppathi, Chia-Her Lin

**Affiliations:** 1 Department of Chemistry, Chung-Yuan Christian University, Chungli District, Taoyuan City 32023, Taiwan; 2 Department of Chemistry, National Taiwan Normal University, Taipei 11677, Taiwan; 3 Department of Biochemical Science and Technology, National Taiwan University, Taipei 10617, Taiwan; thiru9095@gmail.com

**Keywords:** metal-organic framework, nitrogen-doped porous carbon, carbonization, tuning pore size, CO_2_ capture, H_2_O_2_ electrochemical sensor

## Abstract

Nitrogen-doped porous carbon (NPC) materials were successfully synthesized via a Zn-containing metal-organic framework (Zn-MOF). The resulting NPC materials are characterized using various physicochemical techniques which indicated that the NPC materials obtained at different carbonization temperatures exhibited different properties. Pristine MOF morphology and pore size are retained after carbonization at particular temperatures (600 °C-NPC_600_ and 800 °C-NPC_800_). NPC_800_ material shows an excellent surface area 1192 m^2^/g, total pore volume 0.92 cm^3^/g and displays a higher CO_2_ uptake 4.71 mmol/g at 273 k and 1 bar. Furthermore, NPC_600_ material displays good electrochemical sensing towards H_2_O_2_. Under optimized conditions, our sensor exhibited a wide linearity range between 100 µM and 10 mM with a detection limit of 27.5 µM.

## 1. Introduction

Porous carbon materials have been regarded as significant porous materials because of their distinctive properties such as pore size, extraordinary surface area and good electrochemical activities [1,2,3]. They have extensive applications in many fields including catalysis, biosensors, fuel cells and supercapacitors [4,5,6,7,8,9,10,11]. In this sense, 3D porous carbon-based structures are promising to numerous applications, such as contamination removal, gas sorption/separation, and electrode materials [2,12,13]. In particular, CO_2_ capture purpose nitrogen-doped porous carbon (NPC) materials were used, because of its stability, low cost and performance [14,15]. For CO_2_ capture, the pore size of porous material plays a significant role, ultramicropores of ~4 Å to ~8 Å are predominantly suitable for CO_2_ sorption [16,17]. The preparation of porous carbon materials has been synthesized in a known way such as template and activation method [18,19]. Generally, template progression devours a larger quantity of organic or inorganic template and the synthesizing processes are complicated. Activation methods such as KOH and NaOH can afford a high surface area but needed a huge amount of activation agents. To avoid such complication, recently metal-organic framework (MOF) materials have gained tremendous attentions to prepare the porous carbon materials through single step carbonization method. For instance, the recently reported porous carbon material derived from Zn-MOF, having ultramicropore size (~4 Å to ~8 Å) showed excellent CO_2_ capture properties [20]. Since MOFs have gained tremendous attention due to their diverse structures with tunable pore shapes, sizes, volumes, and surface chemistry. Therefore, MOFs have prospective applications in gas storage/separation, electronic devices, chemical sensors, catalysis, and biomedical applications [21,22,23,24,25,26]. The multifunctional MOFs are often chosen to synthesis porous carbon materials; MOF-templated straightforward synthesis provided the high-quality nanoporous carbon with a well-ordered pore size and significant surface area. The morphology of nanoporous carbon can be tuned by optimizing the carbonization method (such as temperature, time, and atmosphere) [1,27,28]. Recently, MOF-derived metal/metal oxide embedded porous carbon materials [29,30] are used in the electrodes for electrochemical sensors [31,32]. But, maintaining the pristine MOF morphology of the resulting porous materials from the carbonization process is a difficult task due to the shrinkage of framework/decomposing organic ligand during carbonization, therefore, a systematic study is necessary [33].

Additionally, hydrogen peroxide (H_2_O_2_) is a toxic oxidizing agent, being used in various fields such as biomedical science, environmental science, food, textile and chemical industries [34,35,36]. Therefore, H_2_O_2_ determination is of practical importance for both environmental and industrial purposes. It has been well established that Zn based material modified electrodes are used for H_2_O_2_ detection [37,38].

**Scheme 1:**
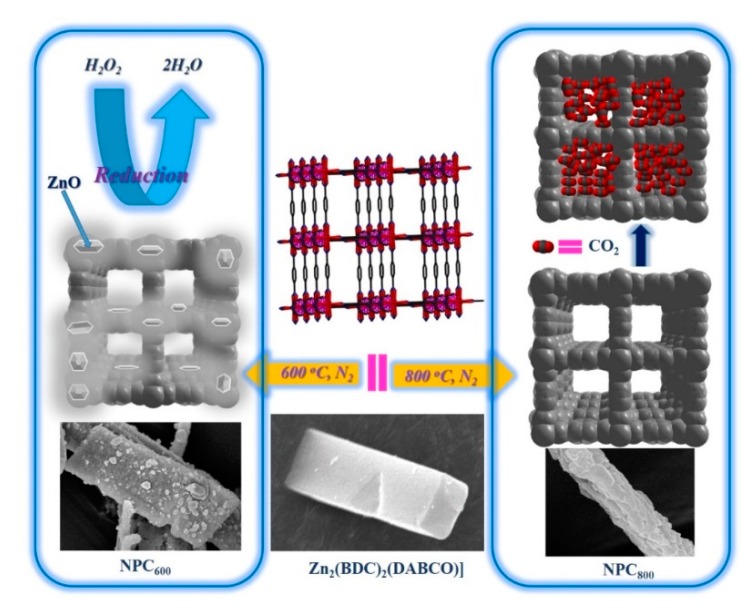
Synthesizing pathway of NPC materials from Zn-MOF.

Therefore, herein, we reported the preparation of NPC materials from {Zn_2_(BDC)_2_(DABCO)} (Zn-MOF) [11] at various temperatures under a N_2_ atmosphere (Scheme 1). The properties of the resulting NPC materials such as morphology, pore size, pore-volume, CO_2_ uptake and H_2_O_2_ electrochemical sensing were investigated.

## 2. Methods 

### 2.1. Materials

Zn(NO_3_)_2_·6H_2_O, (98%) and H_2_BDC, (98%) were bought from Sigma-Aldrich (Burlington, MA, USA), DMF, (≥99.8%) was purchased from Merck (Darmstdt, Germany), (DABCO, 98%) was purchased from Alfa Aesar (Lancashire, UK). All other compounds used throughout this study were of an analytical grade. The electrochemical experiments were performed using a three-electrode system-CHI model 824B workstation with a screen printed carbon electrode (SPCE)/chemically modified SPCE as a working electrode, Ag/AgCl (in 3 M KCl) as a reference electrode, and Pt wire as an auxiliary electrode. SPCE was purchased from Zensor R&D (Taichung, Taiwan). A phosphate buffer solution (0.1 M, pH 7 PBS) was prepared by mixing 0.1 M, NaH_2_PO_4_, and Na_2_HPO_4_. To compare the various electrodes performance, 5 mM of FeCN solution used as a probe. Briefly, the FeCN solution was prepared using 5 mM of K_3_[Fe(CN)_6_] and K_4_[Fe(CN)_6_], and 0.1 M, KCl used as a supporting electrolyte. For H_2_O_2_ detection, 100 mM of H_2_O_2_ prepared from 9.8 M of H_2_O_2_ (30 wt%).

### 2.2. Preparation of Zn-MOF, {Zn_2_(BDC)_2_(DABCO)}

The Zn-MOF was prepared [39] by, a mixture of Zn(NO_3_)_2_·6H_2_O (5.41 mmol, 1.609 g), H_2_BDC (5 mmol, 0.83 g), DABCO (2.5 mmol, 0.28 g) in DMF (60 mL) was taken out into a Teflon-lined autoclave and heated to 120 °C, 2 days later cooling down to room temperature. The Zn-MOF was washed with DMF and dried at room temperature overnight. Further this material was used for the carbonization process.

### 2.3. Preparation of NPC Materials

The NPC materials were prepared through a single-step carbonization method [31]. The 0.400 g of Zn-MOF was transferred into a silica crucible boat and then placed in a furnace chamber. The NPC materials obtained at target temperatures (500, 550, 600, 700, 800, or 900 °C) under an N_2_ atmosphere for a 5 h duration. The resulting materials obtained at 500, 550, 600, 700, 800, and 900 °C are assigned as NPC_500_, NPC_550_, NPC_600_, NPC_700_, NPC_800_ and NPC_900_ respectively.

### 2.4. Electrode Preparation

Prior to electrode modification, the bare SPCE was precleaned electrochemically by potential cycling between −1 and +1 V vs. Ag/AgCl for 6 cycles in 0.1 M pH 7 PBS. NPC_T_ modified SPCE (SPCE/NPC_T_) was prepared by the following procedure: 10 µL of 2000 ppm (2 mg mL^−1^) respective NPC_T_ dispersed in acetonitrile suspension was drop coated on precleaned SPCE, and allowed for dry on the hot plate at 40 °C for 15 min. The NPC_T_ prepared by carbonization under the increasing temperature of 500, 550, 600, 700, 800 and 900 °C. The corresponding modified electrode designated as SPCE/NPC_500_, SPCE/NPC_550_, SPCE/NPC_600_, SPCE/NPC_700_, SPCE/NPC_800_, SPCE/NPC_900_, respectively.

### 2.5. Characterization

The purity of MOF and NPC materials were investigated by powder x-ray diffraction (PXRD) using a Bruker D8 advance instrument (Billerica, MA, USA) equipped with CuKα radiation (*λ* = 1.54178 Å). The morphology of MOF and NPC materials were observed by high-resolution scanning electron microscopy (HR-SEM, JEOL JEM-7600F instrument, Akishima, Japan). The NPC materials morphology was characterized by transmission electron microscopy (TEM, using a JEM-2010 instrument, Tokyo, Japan) at a voltage of 200 KV. The synthesized NPC materials were also recorded with a Raman spectra on a CCD detector (Stanford Computer Optics Inc., Berkeley, CA, USA) using a He-Ne laser with an excitation wavelength of 632.8 nm. The Zn element presence was investigated by inductively coupled plasma-mass spectrometry (ICP-MS, Japan Agilent 7500ce, Tokyo, Japan). The elemental analysis (C, N, O) was executed by an elementar vario EL III CHN-OS elemental analyzer (Germany). N_2_ gas adsorption, CO_2_ gas adsorption of all materials were measured using micrometrics (Norcross, GA, USA) and the gas sorption analysis purpose, the materials were dried at 120 °C for 12 h under vacuum.

## 3. Results and Discussion

### 3.1. Structure, Morphology, and Composition of NPC Materials

**Figure 1:**
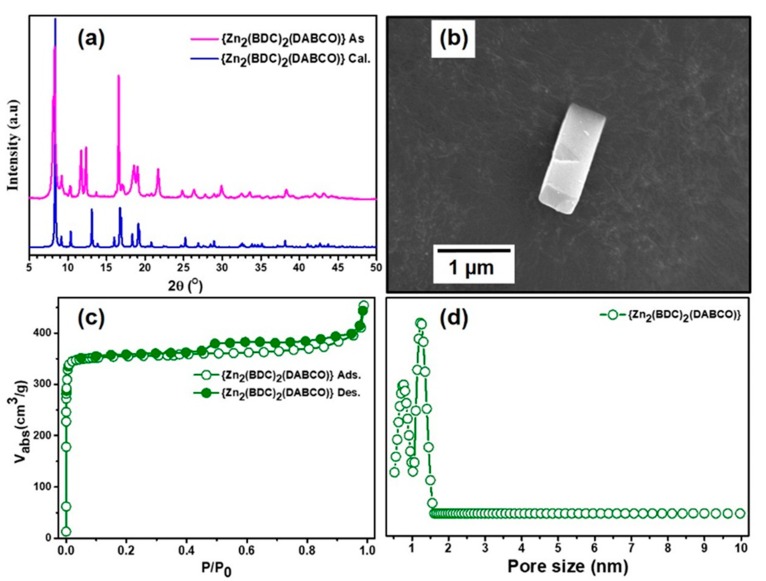
(**a**) PXRD patterns of as-synthesized Zn-MOF, (**b**) SEM image of Zn-MOF (**c**) N_2_ sorption analysis and (**d**) NLDFT pore size distribution profile.

**Figure 2:**
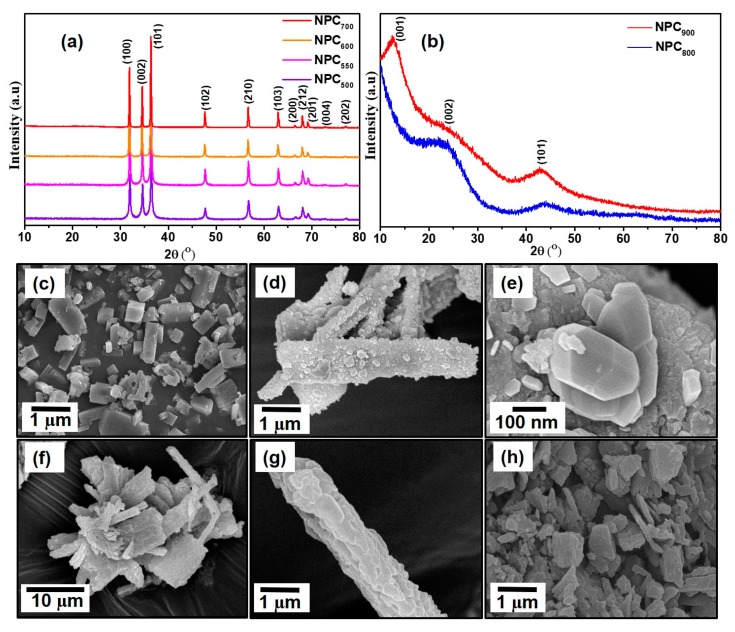
(**a**,**b**) PXRD patterns NPC samples. SEM images of, (**c**) synthesized Zn-MOF at the 1 µm scale (before carbonization), (**d**–**h**) Zn-MOF after carbonization at different temperature. (**d**) NPC_600_ at the 1 µm scale, (**e**) NPC_600_ at the 100 nm scale, (**f**) NPC_800_ at the 10 µm scale, (**g**) NPC_800_ at the 1 µm scale and (**h**) NPC_900_ at the 1 µm scale.

Synthesized Zn-MOF structure and porous properties was checked by PXRD, SEM and N_2_ gas sorption measurements (Figure 1a–d). As expected, the synthesized MOF showed a well-defined crystallinity and surface area of 1700 m^2^/g, and good agreement with the literature [40]. The pore size of Zn-MOF was calculated by the NLDFT method, it reveals two micropores (0.75 and 1.4 nm) (Figure 1d). The SEM images revealed the particle shapes of Zn-MOF was a mixture of the cube, brick, and rod-like shapes (Figure 2c).

The MOF was further exploited to a one-step direct carbonization method to produce the NPC materials. The detailed preparation method is given in Section 2.3. The morphology, crystallinity and surface area of NPC materials were examined by PXRD, SEM, TEM, Raman analysis, N_2_ gas sorption isotherms, ICP-MS, and elemental analysis.

The PXRD patterns of the NPC_500–700_ samples showed the diffraction peaks for the formation of ZnO nanoparticle (Figure 2a). The 2° peaks at 31.7 (100), 34.4 (002), 36.2 (101), 47.4 (102), 56.6 (110), 62.9 (103), 65.5 (200), 68.0 (112) and 69.1 (201) that are lattice planes of ZnO [41]. The NPC_500_, NPC_550_, NPC_700_ morphologies show the shrinking phenomenon of Zn-MOF during carbonization at this particular temperature (Figures S1 and S2). While the morphology retained from pristine MOF, brick, and rod shape at 600 °C (Figure 2d,e and Figure S3). The PXRD pattern of the NPC_800_ sample showed two broad peaks of graphitic carbon at 23 (002) and 44° (101) (Figure 2b) [7]. The absence of ZnO at higher carbonization temperature revealed when the temperature is close to its boiling point of ZnO (907 °C) is reduced to Zn and evaporate.

**Figure 3:**
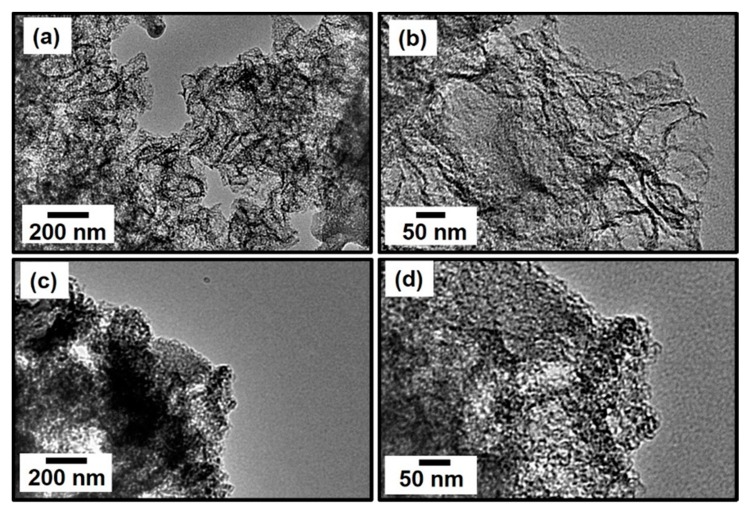
TEM images of (**a**) NPC_800_ at the 200 nm scale, (**b**) NPC_800_ at the 50 nm scale, (**c**) NPC_900_ at the 200 nm scale and (**d**) NPC_900_ at the 50 nm scale.

Furthermore, the SEM revealed the morphology partially retained the pristine MOF with distorted graphitic carbon structures (Figure 2f,g and Figure S4), TEM images noticeably show the presence of an abundant interconnected and oriented multilayer graphene domains can be observed (Figure 3a,b). Further, by increasing the temperature to 900 °C, the PXRD pattern indicated a mixture of graphite oxide (GO) and graphitic carbon. A broad peak at 2θ ≅12 (0 0 1) the reflection of graphite oxide, 23° and 44° crystallographic planes of graphitic carbon, which possess the amorphous carbon structure. SEM (Figure 2h and Figure S5) and TEM (Figure 3c,d) images (900 °C) shown are revealed the pristine MOFs have fully or partially cracked the shapes and the shrinkage of the whole framework.

Further, synthesized NPC materials were characterized by Raman spectroscopy for the degree of graphitization. The spectrum analyzed range between 1200 cm^−1^ to 1700 cm^−1^ bands were fitted with the spectra, 1180 cm^−1^ (A_1_ band), 1340 cm^−1^ (D band), and 1600 cm^−1^ (G band) [42]. Figure S6 shows significantly broadened D and G bands. Gaussian fitting was used to separate the A_1_, D, G band and fitted after baseline subtraction. The I_D_/I_G_ ratio increased while increasing the temperature, indicating the formation of disorder with a low degree of graphitization of NPC materials was obtained. The I_D_/I_G_ ratio between D and G bands revealed the degree of graphitization in carbon-related materials. Temperature 500–700 °C carbonized materials obtained a higher degree of graphitization, due to the ZnO present in the carbon material. Because there is still a definite chemical interaction between ZnO and N atoms, the redshifts of the D bands by approximately 15 cm^−1^ to ≅1326 were observed [43]. Further, as we increased the temperature (800 and 900 °C), ZnO-N adducts were not detected and were also evidenced by PXRD. As the ratio is higher, it could be a low degree of graphitization, particularly those materials carbonized at ≥800 °C (see Table 1). The G band shifted to higher frequencies by approximately 6 cm^−1^ due to the nitrogen present in the NPC materials [44]. Chemical compositions of NPC were studied by ICP-MS and elemental analysis (Figure S7). The zinc contents were investigated by ICP-MS, increasing carbonization temperature results the decreasing zinc percentage are 51.7% (600 °C), 43.72% (700 °C), 4.95% (800 °C), 0.26% (900 °C) and an appreciable amount of nitrogen (1.96–2.98 wt%) based on elemental analysis.

### 3.2. Porous Property and CO_2_ Uptake of NPC Materials

**Figure 4:**
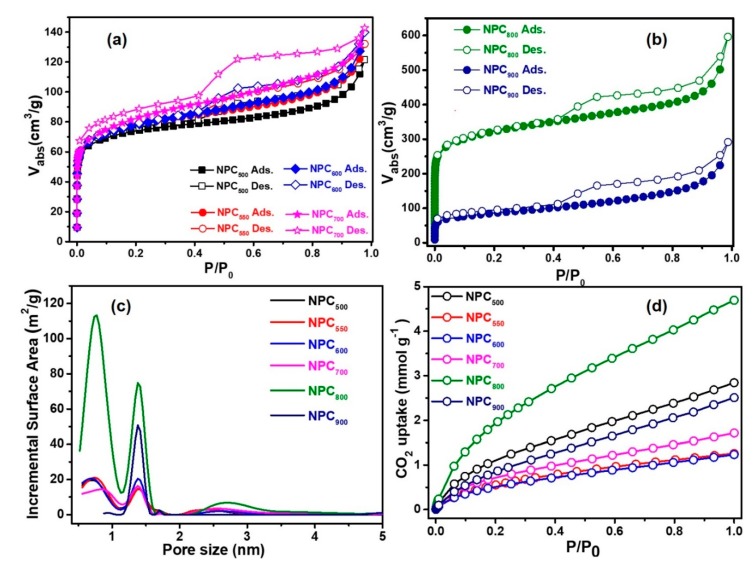
(**a**,**b**) N_2_ sorption analysis at 77 K, (**c**) NLDFT pore size distribution profile and (**d**) CO_2_ sorption isotherm at 273 K and 1 bar of NPC materials.

The textural properties of these NPC materials were evaluated by the N_2_ sorption analyzer. The Figure 4a–c represented the N_2_ uptake isotherm and corresponding pore sizes of the NPC materials. Table 1 represents the NPC material’s surface area, pore volume and pore size. The N_2_ sorption curves of the NPC materials possess type-I isotherms that steeply climb in the low-pressure range (P/P_0_ = 0−0.10), suggesting that micropores were dominant [45]. In the high-pressure range (P/P_0_ = 0.40−1.00), there were decent increases in the adsorption in all samples and a slight hysteresis loop between the sorption curves, which revealed that mesopores were also present in the materials. The surface area and total pore volume of the NPC_500–700_ materials is nearly equal to 273, 287, 289, 296 m^2^/g and 0.18, 0.20, 0.22, 0.22 cm^3^/g correspondingly (see Table 1).

Whereas the BET surface area of the NPC_800_ material has 1192 m^2^/g and a total pore volume 0.92 cm^3^/g. The majority of the pores of NPC_500–800_ materials are 0.75, 1.4 nm retained from the pristine MOF (Figure 4c), while there are mesopores around 2.1–3 nm, which specifies the occurrence of mesopores in the NPC materials (Figures S8–S13). While the NPC_900_ materials show a lesser surface area (303 m^2^/g), pore volume (0.45 cm^3^/g) indicates framework shrinkage and fragmentation throughout the high-temperature carbonization process. The total pore volume of NPC_800_ material has a better percentage than the NPC_500–700_ and NPC_900_ materials. It is noted that the NPC_800_ material reached 42% of micropores, whereas, the percentage of V_micro_/V_total_ decreased significantly to 13% in the NPC_900_ sample. CO_2_ sorption is investigated for the NPC materials at 273 K at 1 bar (Figure 4d). NPC_800_ demonstrated a higher CO_2_ capture of 4.71 mmol/g than NPC_500_ (2.85 mmol/g), NPC_550_ (1.20 mmol/g), NPC_600_ (1.24 mmol/g), NPC_700_ (1.71 mmol/g) and NPC_900_ (2.51 mmol/g) at 273 K and 1 bar. Such a micro-mesoporous structure of NPC_800_ material provides a fast diffusion of CO_2_ into the inner pores material. NPC_800_ material showed CO_2_ capacity value closely matches/ greater than those of the carbon-related materials (see Table S1).

### 3.3. Comparisons Voltammetric Behavior of Various SPCE/NPC Modified Electrode in FeCN

**Figure 5:**
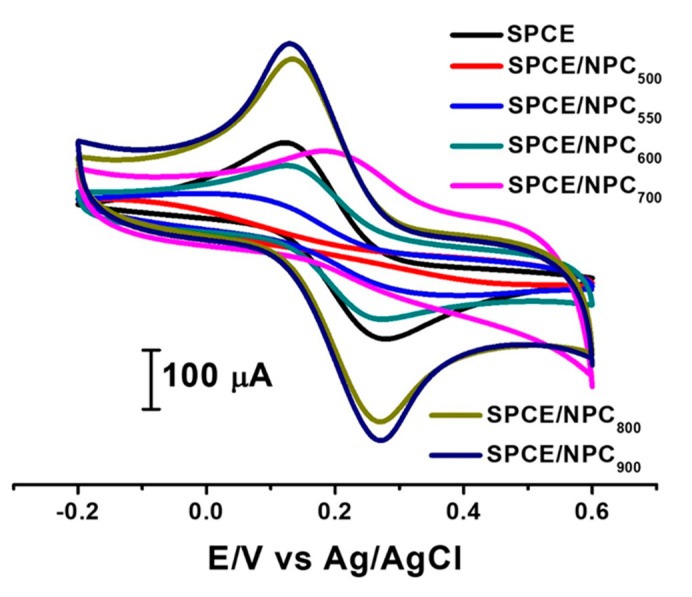
Cyclic voltammetric response of SPCE and SPCE/NPC_T_ in 5 mM ferric cyanide solution.

CV analysis was executed to study the electrochemical behavior of SPCE and SPCE/NPC_T_ modified electrodes in 5 mM FeCN under a potential window from –0.2 to +0.6 V. As can be seen in Figure 5, bare SPCE exhibit a well-defined reversible redox peak at E° = +193 mV with a peak to peak potential difference (ΔEp = Epa − Epc) value of 126 mV, which is the characteristic peak for Fe^2+^/Fe^3+^ interconversion [46,47]. After SPCE/NPC_T_ modification, relatively higher/lower redox current responses were noticed with a ΔEp value of about 561, 235, 125, 112, 137 and 140 mV, while the relative current change (ΔIa) was recorded for SPCE/NPC_500_, SPCE/NPC_550_, SPCE/NPC_600_, SPCE/NPC_700_, SPCE/NPC_800_, SPCE/NPC_900_ of about −99, −88, −29, −12, +83 and +113 µA, respectively. The observation is due to the semiconductor Zn moieties existing up to a carbonization temperature of 700 °C that results in a decrease in FeCN signal. In contrary, carbonization at 800 and 900 °C produced relatively smaller Zn moieties with NPC and hence an increase in signal. This result suggests that the semiconductor Zn content decreased with increasing carbonization temperature. In other words, the FeCN current response is inversely proportional to the Zn content. The obtained results coincide with the elemental analysis, ICP-MS and PXRD results.

### 3.4. Detection of H_2_O_2_ at SPCE/NPC_T_

**Figure 6:**
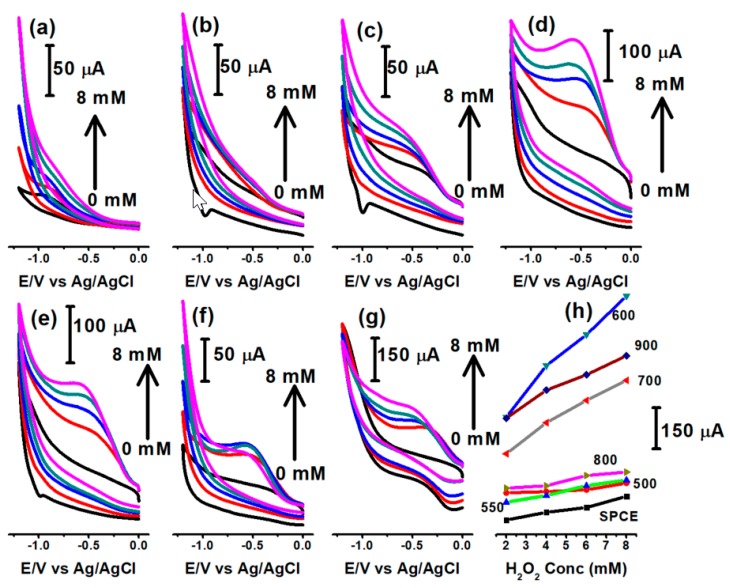
Cyclic voltammetric detection of H_2_O_2_ in 0.1 M, pH 7PB (**a**) SPCE, (**b**) SPCE/NPC_500_, (**c**), SPCE/NPC_550_, (**d**) SPCE/NPC_600_, (**e**) SPCE/NPC_700_, (**f**) SPCE/NPC_800_ and (**g**) SPCE/NPC_900_, respectively. (**h**) Their corresponding current verses concentration plots.

The surface area and porous defective sites have an important role in the electrochemical sensors. Therefore, H_2_O_2_ sensing applicability was tested for SPCE/NPC_500_, SPCE/NPC_550_, SPCE/NPC_600_, SPCE/NPC_700_, SPCE/NPC_800_, SPCE/NPC_900_, respectively. Figure 6 shows electro catalytic reduction CVs of H_2_O_2_ at various SPCE/NPC_T_ electrodes. CV measurements were done in 0.1 M, pH 7.4 PB solution under the potential sweeping from 0 to −1.2 V at a scan rate of 100 mV s^−1^. During the cathodic segment, H_2_O_2_ reduction peak [48] was noticed at ~−0.7 V for SPCE and for SPCE/NPC_T_ the same reduction peak was noticed with lower over potential (~−0.4 V). Among the various electrodes, detection response are clearer and more explicit at SPCE/NPC_600_. These results evidently exposed that the SPCE/NPC600 electrode exhibited better electrocatalytic H_2_O_2_ reduction than other electrodes. Therefore, SPCE/NPC_600_ was chosen for further studies.

### 3.5. Flow Injection Analysis (FIA) Detection of H_2_O_2_ at SPCE/NPC_600_

**Figure 7:**
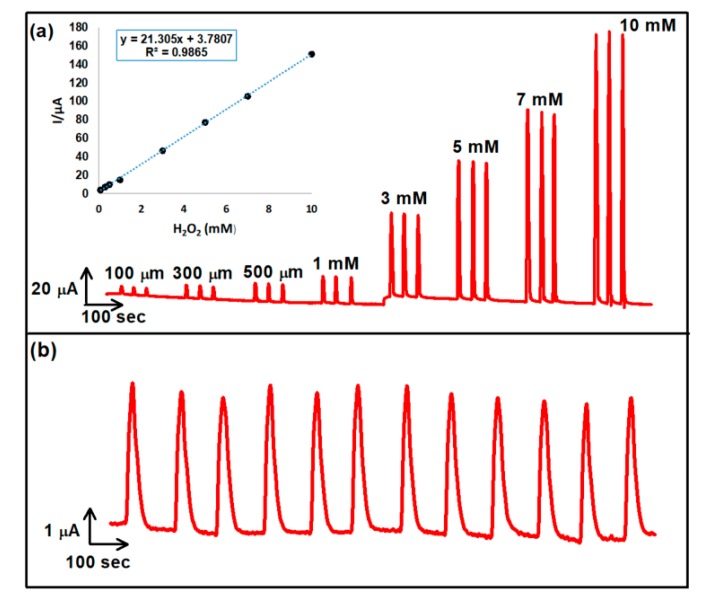
(**a**) Detection of H_2_O_2_ by FIA method at SPCE/NPC_600_, (**b**) FIA responses of 12 continuous injections of 0.5 mM H_2_O_2_.

The above observation was further utilized for amperometric FIA analysis of H_2_O_2_. The H_2_O_2_ reduction current increased linearly with increasing concentration, in which H_2_O_2_ was electrochemically reduced at −0.4 V by applying a potential, and thus yielded quantitative current responses corresponding to the content of H_2_O_2_ (Figure 7). A wide linearity range between 100 μM and 10 mM with a R^2^ value of 0.9865 and a limit of detection (LOD) 27.5 μM were obtained. In order to access the repeatability of a SPCE/NPC_600_ modified electrode, 12 repeated injections of 0.5 mM H_2_O_2_ were performed and a RSD value of 4.13% was obtained. Compared to a few other Zn based H_2_O_2_ sensors (Table S2), the present method exhibited a wide linear range along with a specific sensitivity of 108.7 μA mM^−1^ cm^−2^.

## 4. Conclusions

Herein, we reported the synthesized NPC materials at various carbonization temperatures at a constant time under a N_2_ atmosphere. NPC materials surface, morphology, chemical composition, porous properties where characterized by PXRD, SEM, TEM, Raman spectroscopy, ICP-MS, elemental analysis, and 77 K N_2_ sorption isotherms. Pristine MOF pore size was tuned to the porous carbon material, such an ultramicropore, micropore and mesopore combined in unique material and interaction between ZnO to NPC to give the pathway to synthesize the effective electrochemical property and CO_2_ sorption materials. These combinations in NPC_800_ exhibited a higher CO_2_ uptake of 4.71 mmol g^−1^ compare to other NPC_T_ materials. NPC_600_ displayed a good electrochemical reduction towards H_2_O_2_. Under optimal conditions, our sensor exhibited linearity that ranged from 0.1–10 mM, which confirmed its sensitive response to H_2_O_2_ over a wide range of concentrations. The detection limit was determined to be 27.5 µM (S/N = 3)

## Figures and Tables

**Table 1:** Surface and porous properties of the NPC samples.

Sample	I_D_/I_G_	S_BET_*^a^* (m^2^/g)	V*_total_^b^* (cm^3^/g)	V*_micro_* *^c^* (cm^3^/g)	Pore Size (nm)	CO_2_ uptake*^d^* (mmol/g) (wt %)
NPC_500_	0.92	273	0.18	0.09 (50)	0.75, 1.4, 2.1~3	2.85 (12.54)
NPC_550_	0.98	287	0.20	0.091 (45)	0.75, 1.4, 2.1~3	1.20 (5.28)
NPC_600_	1.01	289	0.22	0.094 (42)	0.75, 1.4, 2.1~3	1.24 (5.46)
NPC_700_	1.07	296	0.22	0.10 (45)	0.89, 1.4, 2.1~3	1.71 (7.52)
NPC_800_	1.24	1192	0.92	0.39 (42)	0.75, 1.4, 2.1~3	4.71 (20.72)
NPC_900_	1.25	303	0.45	0.06 (13)	1.4, 5-10	2.51 (11.04)

*^a^* S_BET_ surface area was examined in the P/P_0_ range of 0.01 to 0.1, which gave the best linearity. *^b^* Total pore volume at P/P_0_ = 0.99. *^c^* Micropore volume (≤ 2 nm) and the values in asides are the percentage of the micropore volume relative to the total pore volume (V*_micro_*/V*_total_*). *^d^* CO_2_ uptake at 273 K and 1 bar and the values in asides are weight percentage (wt%).

## Supplementary Materials

The following are available online at https://www.mdpi.com/1996-1944/13/2/264/s1, Figure S1. SEM image of (a) NPC_500_ and (b) NPC_550_. Figure S2. SEM image of NPC_700_ (a,b). Figure S3. SEM images of NPC_600_ (a–d). Figure S4. SEM image of NPC_800_ (a–d). Figure S5. SEM image of NPC_900_ (a–d). Figure S6. Raman spectra of the obtained NPC materails. (a) NPC_500_, (b) NPC_550_, (c) NPC_600_, (d) NPC_700_, (e) NPC_800_ and (f) NPC_900_. Figure S7. Relative atom percentage at different carbonization temperature (600–900 °C). Figure S8. NLDFT pore size distribution profile for NPC_500_. Figure S9. NLDFT pore size distribution profile for NPC_550_. Figure S10. NLDFT pore size distribution profile for NPC_600_. Figure S11. NLDFT pore size distribution profile for NPC_700_. Figure S12. NLDFT pore size distribution profile for NPC_800_. Figure S13. NLDFT pore size distribution profile for NPC_900_. Table S1. Comparison of CO_2_ uptake with previously reported carbon related materials and MOF-derived carbon materials at temperature 273K in 1 bar. Table S2. Comparison of Zn electrode-based H_2_O_2_ sensors with previously reported ZnO/carbon related materials.
