# Supplementary material for: Carbonization and Preparation of Nitrogen-Doped Porous Carbon Materials from Zn-MOF and Its Applications
Source: Materials (Basel). 2020 Jan 7;13(2):264. doi: 10.3390/ma13020264 (PMC7013983; doi:10.3390/ma13020264)
Supplement: Supplementary file 1 [file materials-13-00264-s001.pdf]

materials

Supplementary

Carbonization and Preparation of Nitrogen-Doped Porous Carbon Materials from Zn-MOF and its Applications

Kulandaivel Sivasankar [1,*], Souvik Pal [2,*], Murugan Thiruppathi [3], Chia-Her Lin [2,*]

[1] Department of Chemistry, Chung-Yuan Christian University, Chungli District, Taoyuan City 32023, Taiwan; sivasankarmpm@gmail.com.

[2] Department of Chemistry, National Taiwan Normal University, Taipei 11677, Taiwan; souvikchem@ntnu.edu.tw; chiaher@ntnu.edu.tw.

[3] Department of Biochemical Science and Technology, National Taiwan University, Taipei 10617, Taiwan; thiru9095@gmail.com.

* Correspondence: sivasankarmpm@gmail.com (K.S); souvikchem@ntnu.edu.tw (S.P); chiaher@ntnu.edu.tw (C-H.L). Tel.: +886-2-77346221 (C-H.L); Fax: +886-2-29324249 (C-H.L).

Received: 26 November 2019; Accepted: 3 January 2020; Published: date

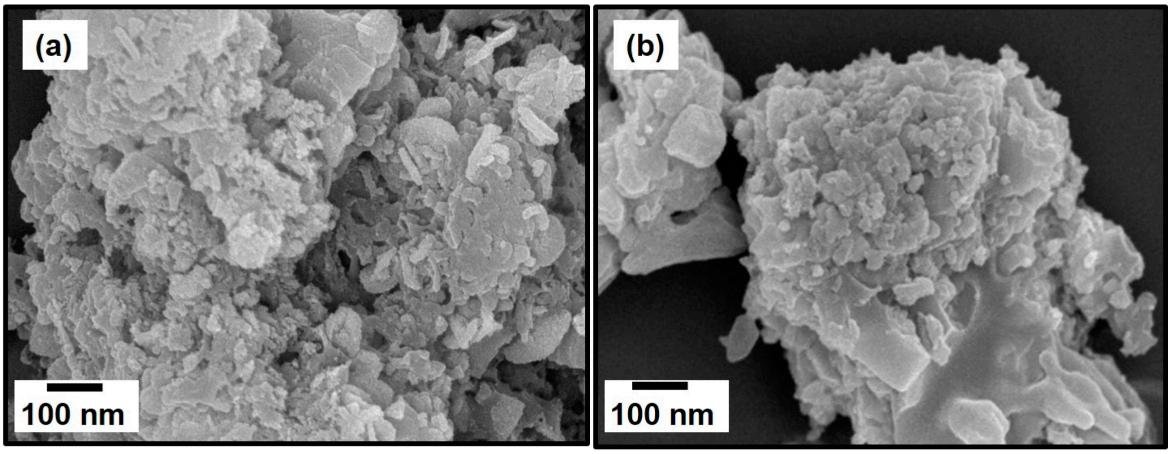

Figure S1. SEM image of NPC_{500} (a) and NPC_{550} (b).

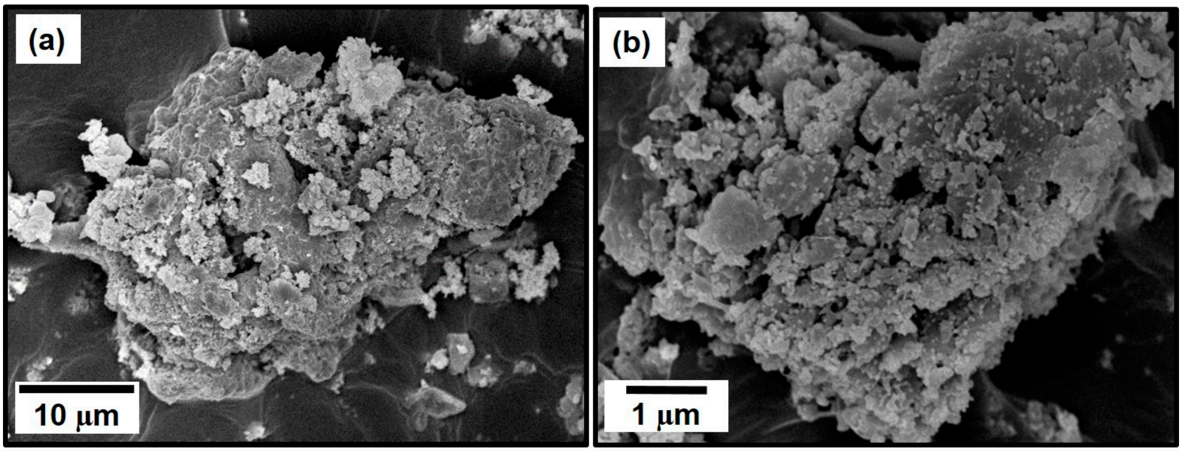

Figure S2. SEM image of NPC_{700} (a,b).

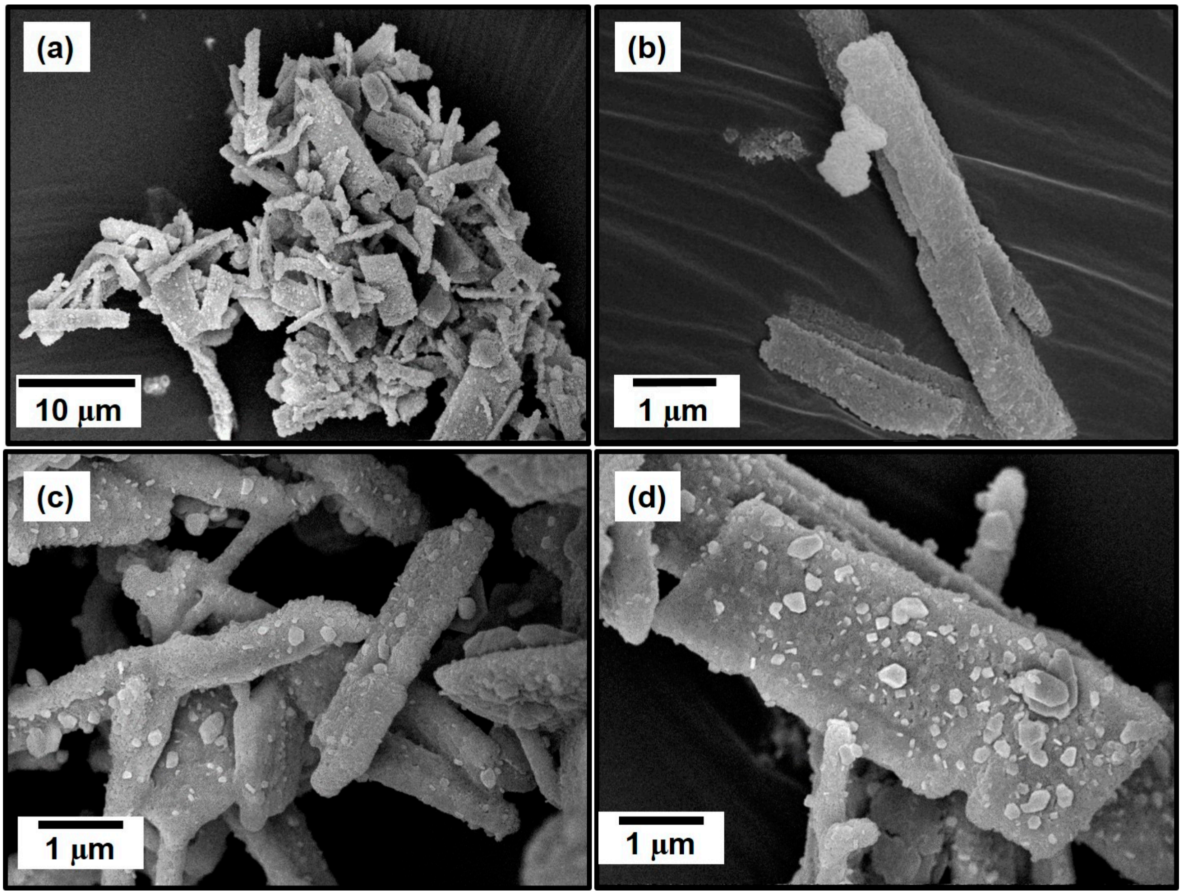

Figure S3. SEM images of NPC_{600} (a–d).

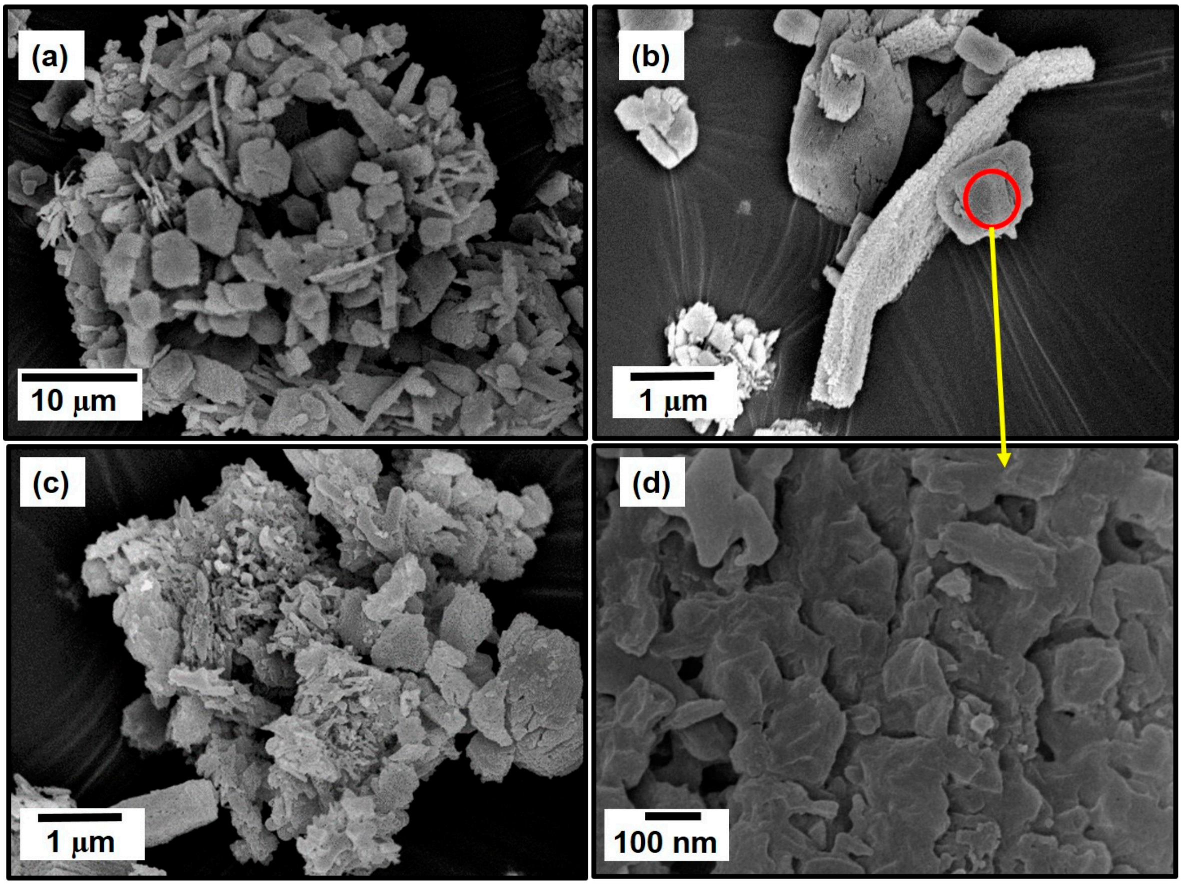

Figure S4. SEM image of NPC_{800} (a–d).

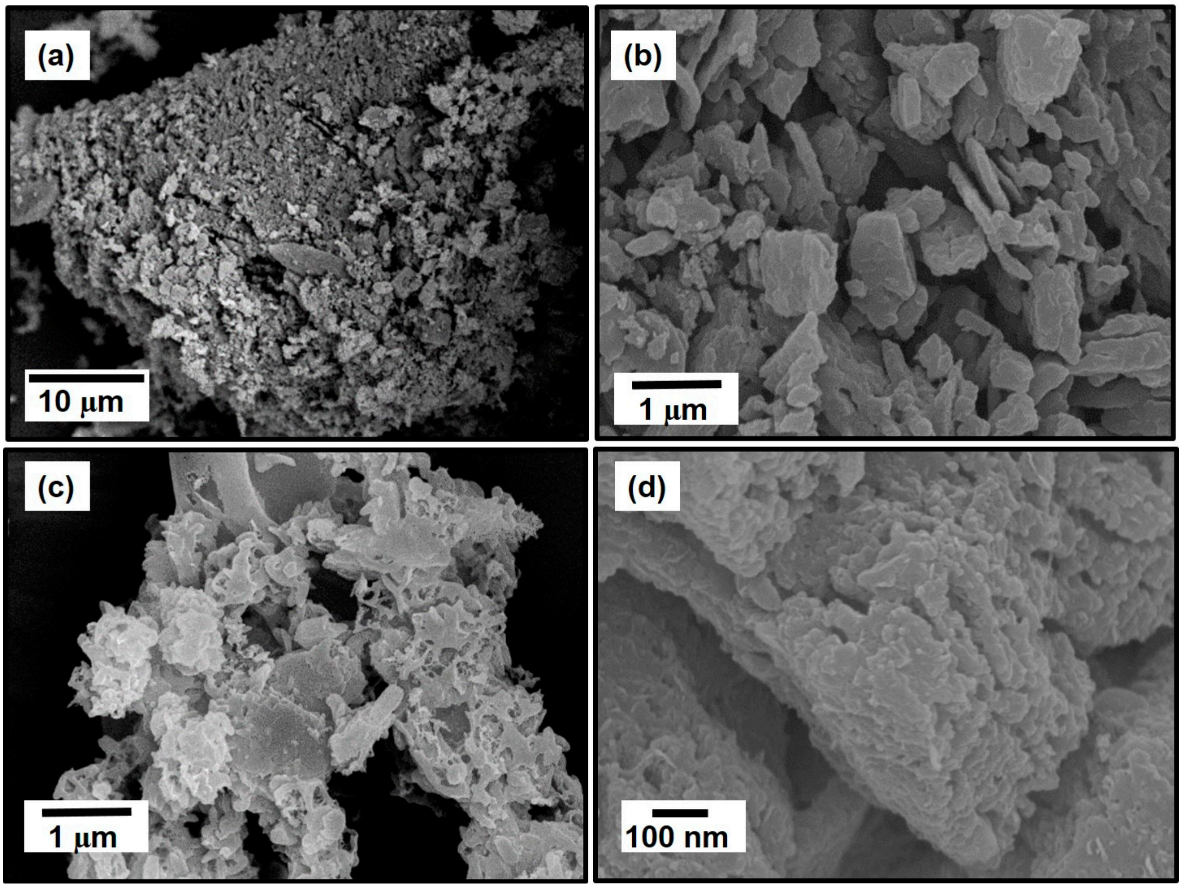

Figure S5. SEM image of NPC_{900} (a–d).

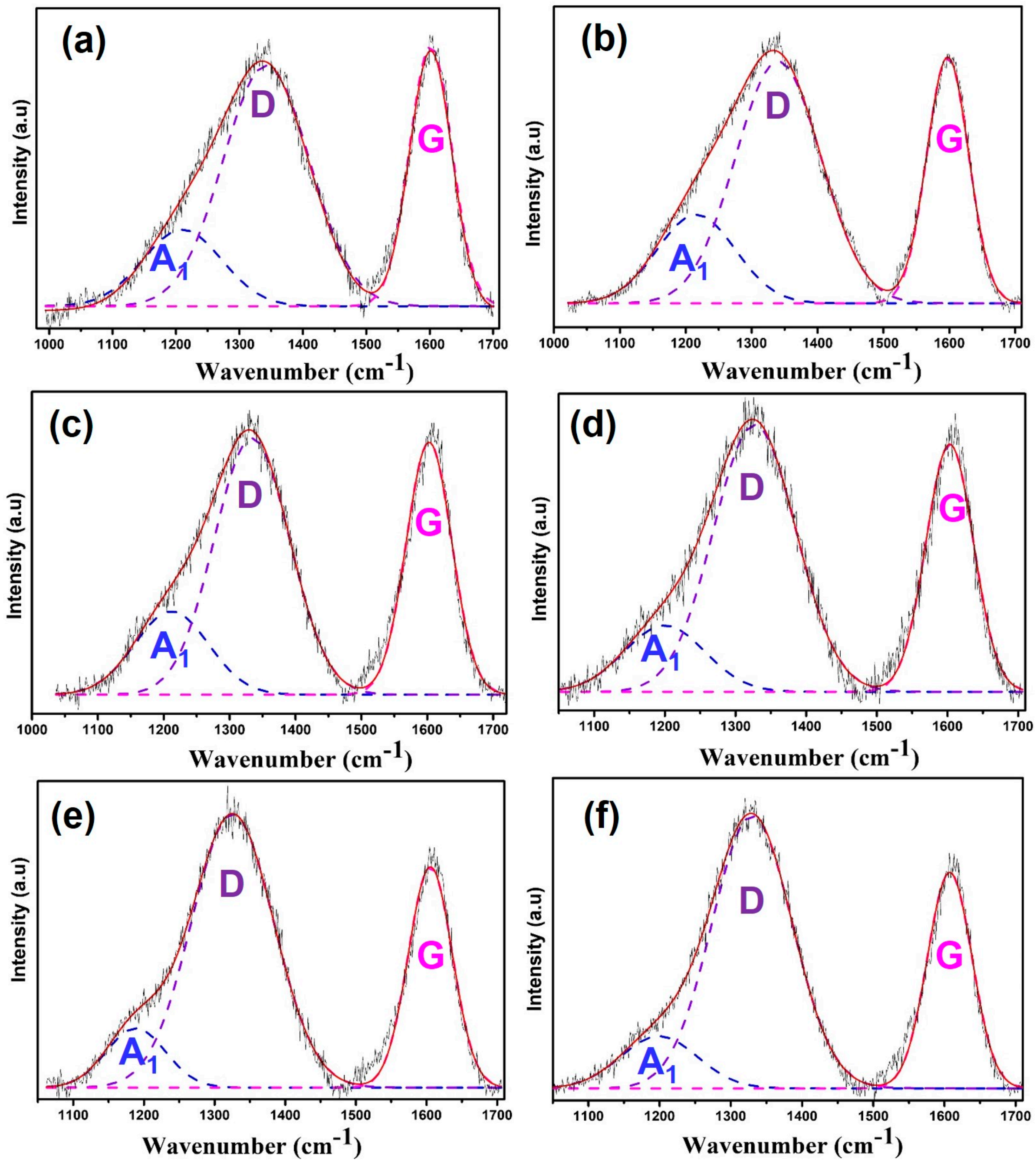

Figure S6. Raman spectra of the obtained NPC materails. (a) NPC_{500}, (b) NPC_{550}, (c) NPC_{600}, (d) NPC_{700}, (e) NPC_{800} and (f) NPC_{900}.

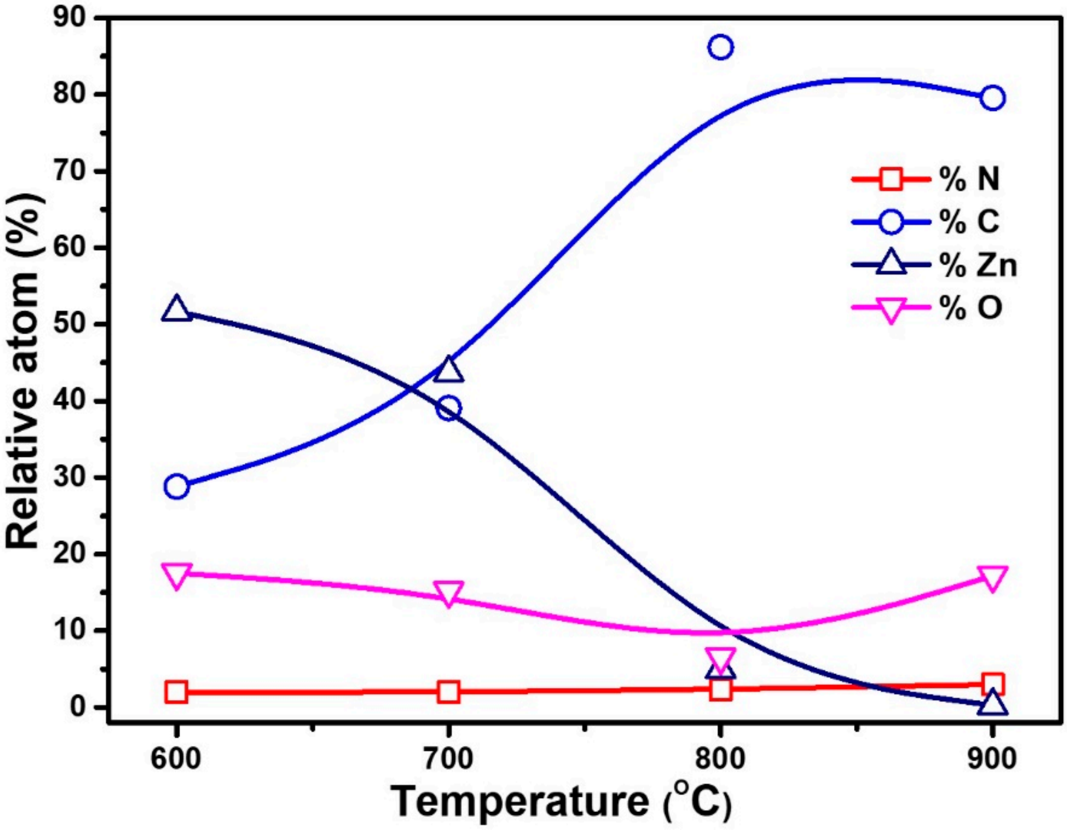

Figure S7. Relative atom percentage at different carbonization temperature (600–900 °C).

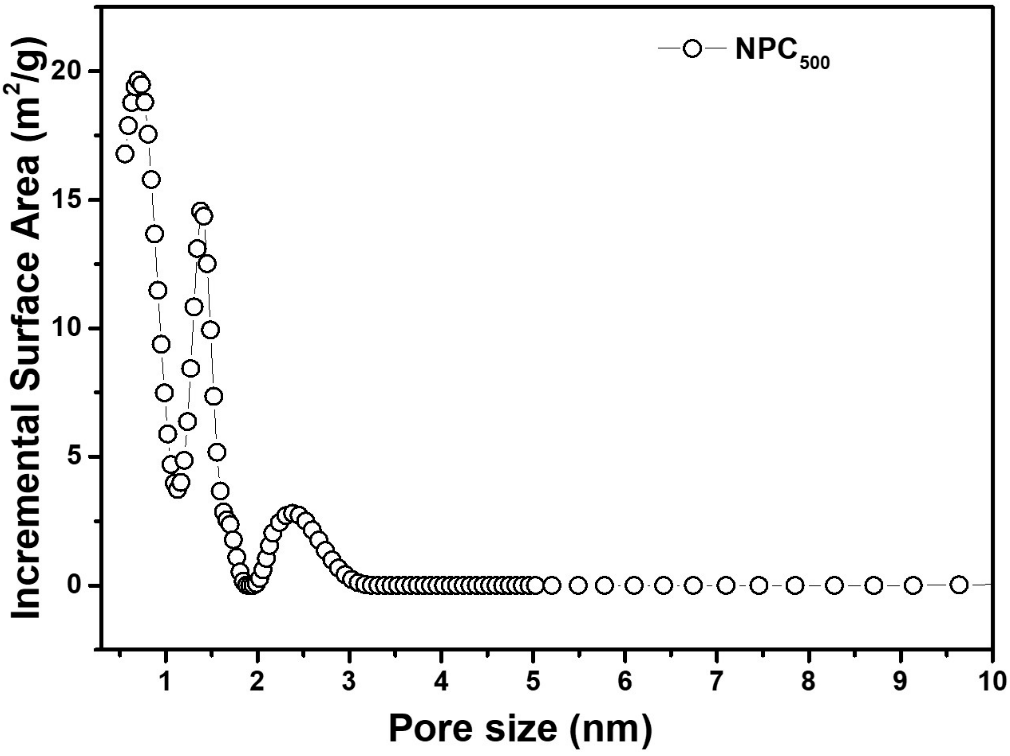

Figure S8. NLDFT pore size distribution profile for NPC_{500}.

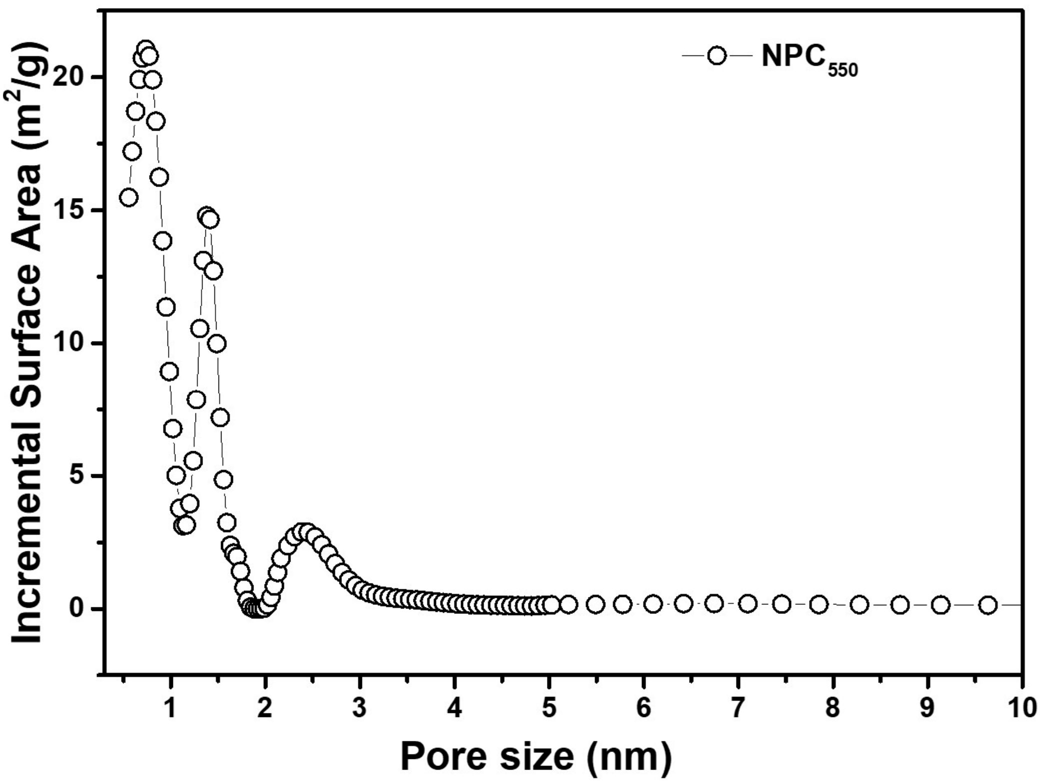

Figure S9. NLDFT pore size distribution profile for NPC_{550}.

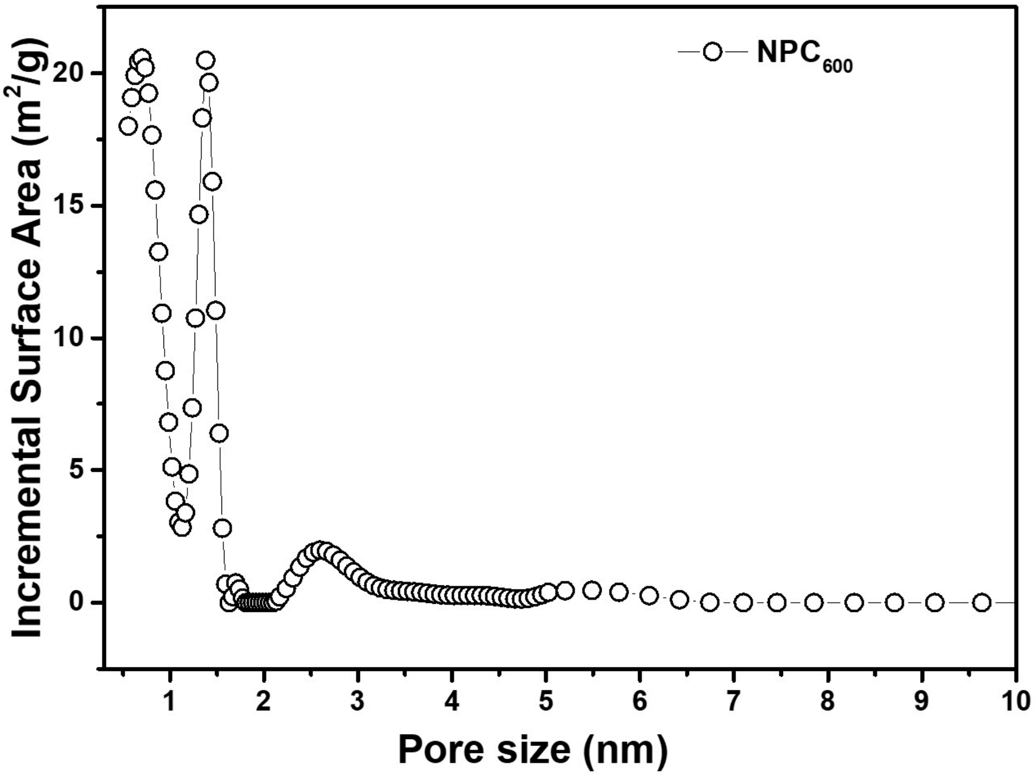

Figure S10. NLDFT pore size distribution profile for NPC_{600}.

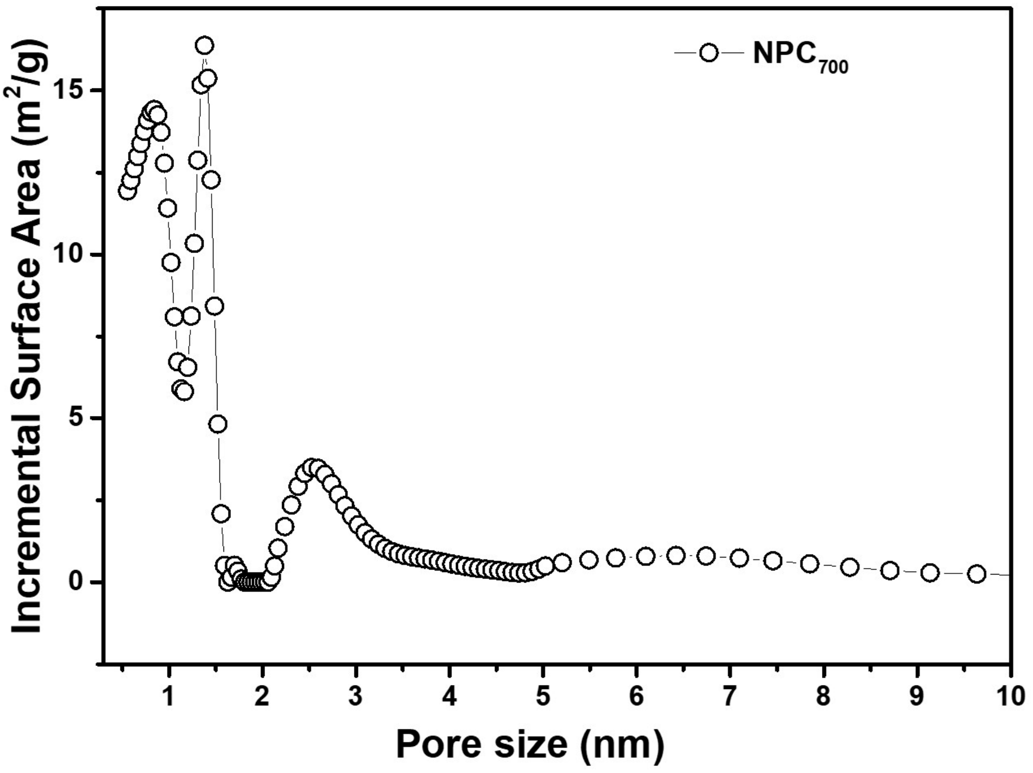

Figure S11. NLDFT pore size distribution profile for NPC_{700}.

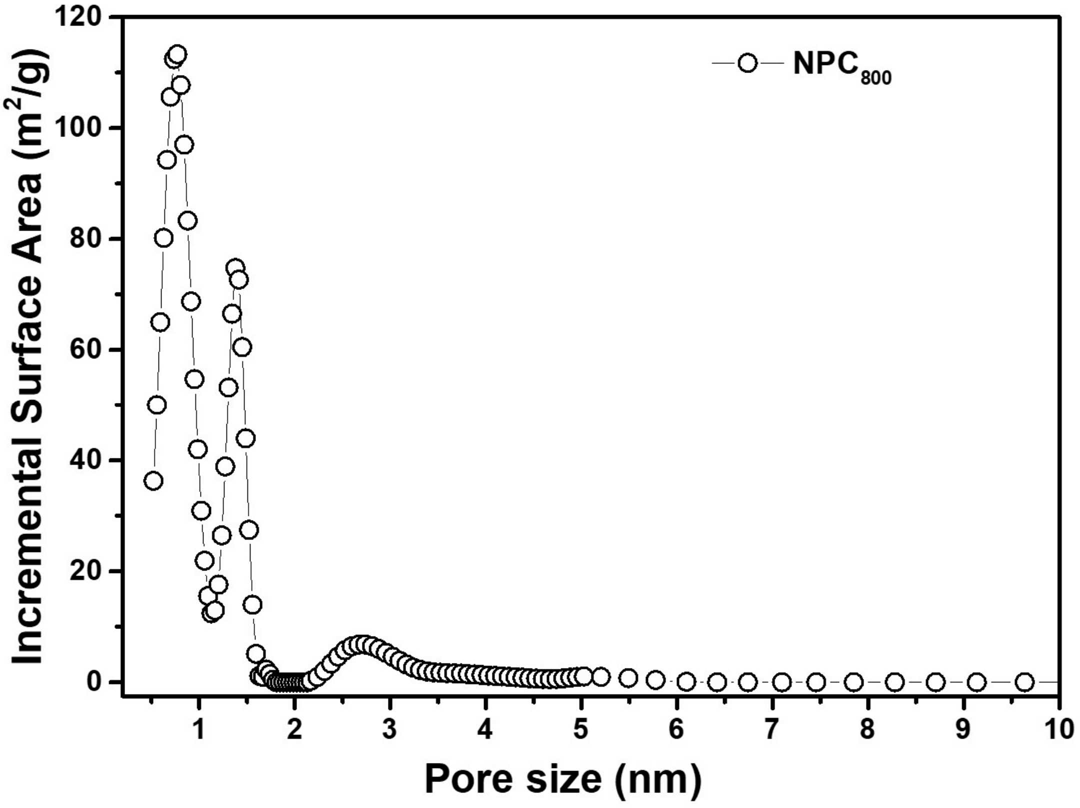

Figure S12. NLDFT pore size distribution profile for NPC_{800}.

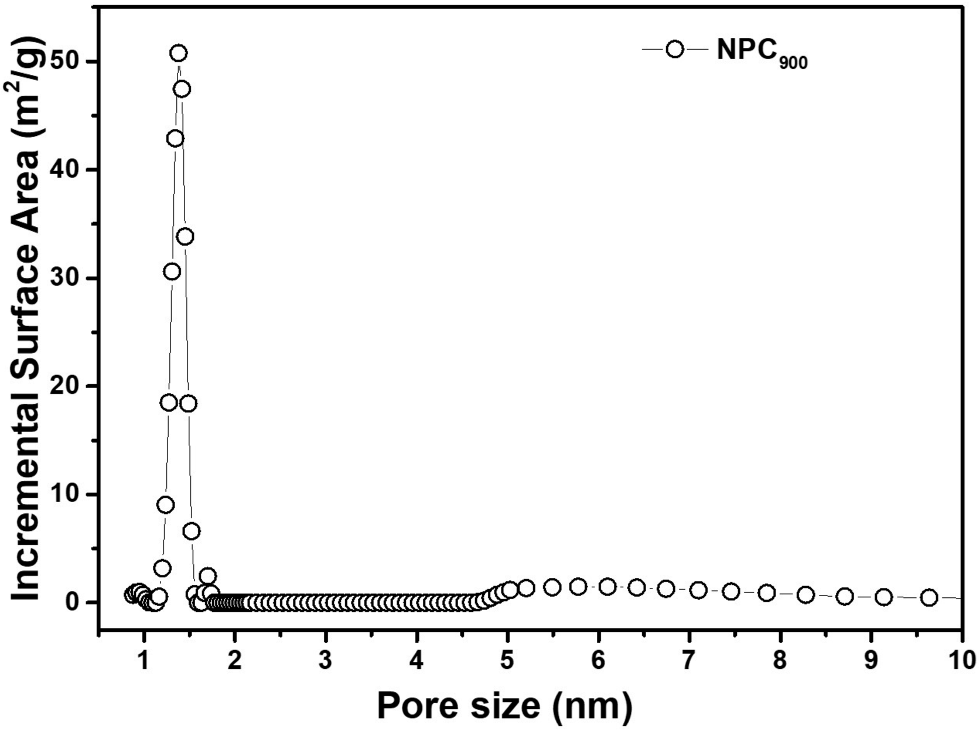

Figure S13. NLDFT pore size distribution profile for NPC_{900}.

Table S1. Comparison of CO_2 uptake with previously reported carbon related materials and MOF-derived carbon materials at temperature 273K in 1 bar.

S. No.	Sample name	CO_2 uptake mmol g^{-1} (wt%)	References
1.	HPG	1.8 (7.92)	[1]
2.	GO-based hydrogel	2.4 (10.56)	[2]
3.	SAGA	2.5 (11.00)	[3]
4.	GO/PET	2.5 (11.00)	[4]
5.	Graphene/terpyridine	2.7 (11.88)	[5]
6.	NDAB3-500	3.6 (15.84)	[6]
7.	IRMOF-3/800	3.99 (17.6)	[7]
8.	NPC-950-100	4.66 (20.5)	[8]
9.	**NPC_{800}**	**4.71 (20.72)**	**At present work**
10.	KBM-700	4.75 (20.9)	[9]

Table S2. Comparison of Zn electrode-based H_2O_2 sensors with previously reported ZnO/carbon related materials.

Electrode material	Detection method	Electrolyte	Linear range	Detection limit	Sensitivity (μA mM^{-1} cm^{-2})	Reference
Co doped ZnO/GCE	CV	pH 7, PB	5-20 mM	14.3 μM	92.45	[10]
Nafion/ZnO/MWNTs/GCE	CV	pH 7.4, PB	1-20 mM	-	-	[11]
Pd/$ZnFe_2O_4$/rGO	Amperometry	pH 7.4, PB	0.025-10.2 mM	2.12 μM	621.64	[12]
SPCE/NPC_{600}	Amperometry	pH 7, PB	0.1-10 mM	27.5 μM	108.7	Present work

References

1. Xia, K.; Tian, X.; Fei, S.; You, K. Hierarchical porous graphene-based carbons prepared by carbon dioxide activation and their gas adsorption properties. *Int. J. Hydrogen Energy* **2014**, *39*, 11047–11054, doi:10.1016/j.ijhydene.2014.05.059.
2. Sui, Z.-Y.; Han, B.-H. Effect of surface chemistry and textural properties on carbon dioxide uptake in hydrothermally reduced graphene oxide. *Carbon* **2015**, *82*, 590–598, doi:10.1016/j.carbon.2014.11.014.
3. Sui, Z.-Y.; Meng, Q.-H.; Li, J.-T.; Zhu, J.-H.; Cui, Y.; Han, B.-H. High surface area porous carbons produced by steam activation of graphene aerogels. *J. Mater. Chem. A* **2014**, *2*, doi:10.1039/c4ta01387e.
4. Sui, Z.Y.; Cui, Y.; Zhu, J.H.; Han, B.H. Preparation of three-dimensional graphene oxide-polyethylenimine porous materials as dye and gas adsorbents. A*CS Appl. Mater. Interfaces* **2013**, *5*, 9172–9179, doi:10.1021/am402661t.
5. Zhou, D.; Cheng, Q.-Y.; Cui, Y.; Wang, T.; Li, X.; Han, B.-H. Graphene–terpyridine complex hybrid porous material for carbon dioxide adsorption. *Carbon* **2014**, *66*, 592–598, doi:10.1016/j.carbon.2013.09.043.
6. Singh, G.; Kim, I.Y.; Lakhi, K.S.; Joseph, S.; Srivastava, P.; Naidu, R.; Vinu, A. Heteroatom functionalized activated porous biocarbons and their excellent performance for CO2 capture at high pressure. *J. Mater. Chem. A* **2017**, *5*, 21196–21204, doi:10.1039/c7ta07186h.
7. Ding, S.; Dong, Q.; Hu, J.; Xiao, W.; Liu, X.; Liao, L.; Zhang, N. Enhanced selective adsorption of CO2 on nitrogen-doped porous carbon monoliths derived from IRMOF-3. *Chem. Commun. (Camb.)* **2016**, *52*, 9757–9760, doi:10.1039/c6cc04416f.
8. Sun, Y.-N.; Sui, Z.-Y.; Li, X.; Xiao, P.-W.; Wei, Z.-X.; Han, B.-H. Nitrogen-Doped Porous Carbons Derived from Polypyrrole-Based Aerogels for Gas Uptake and Supercapacitors. *ACS Appl. Nano Mater.* **2018**, *1*, 609–616, doi:10.1021/acsanm.7b00089.
9. Pan, Y.; Zhao, Y.; Mu, S.; Wang, Y.; Jiang, C.; Liu, Q.; Fang, Q.; Xue, M.; Qiu, S. Cation exchanged MOF-derived nitrogen-doped porous carbons for CO2 capture and supercapacitor electrode materials. *J. Mater. Chem. A* **2017**, *5*, 9544–9552, doi:10.1039/c7ta00162b.
10. Khan, S.B.; Rahman, M.M.; Asiri, A.M.; Asif, S.A.B.; Al-Qarni, S.A.S.; Al-Sehemi, A.G.; Al-Sayari, S.A.; Al-Assiri, M.S. Fabrication of non-enzymatic sensor using Co doped ZnO nanoparticles as a marker of H2O2. *Physica E Low Dimens. Syst. Nanostruct.* **2014**, *62*, 21–27, doi:org/10.1016/j.physe.2014.04.007.
11. Wayu, M.B.; Spidle, R.T.; Devkota, T.; Deb, A.K.; Delong, R.K.; Ghosh, K.C.; Wanekaya, A.K.; Chusuei, C.C. Morphology of hydrothermally synthesized ZnO nanoparticles tethered to carbon nanotubes affects electrocatalytic activity for H2O2 detection. *Electrochim. Acta* **2013**, *97*, 99–104, doi:10.1016/j.electacta.2013.02.028.
12. Ning, L.; Liu, Y.; Ma, J.; Fan, X.; Zhang, G.; Zhang, F.; Peng, W.; Li, Y. Synthesis of Palladium, ZnFe2O4 Functionalized Reduced Graphene Oxide Nanocomposites as H2O2 Detector. Ind. Eng. Chem. Res. **2017**, *56*, 4327–4333, doi:10.1021/acs.iecr.6b04964.

© 2020 by the authors. Submitted for possible open access publication under the terms and conditions of the Creative Commons Attribution (CC BY) license (http://creativecommons.org/licenses/by/4.0/).
